# Supplementary figures and images for: Home health monitoring around the time of surgery: qualitative study of patients’ experiences before and after joint replacement
Source: BMJ Open. 2019 Dec 15;9(12):e032205. doi: 10.1136/bmjopen-2019-032205 (PMC6924768; doi:10.1136/bmjopen-2019-032205)

## Supplementary File 1 – Decision Coding Tree

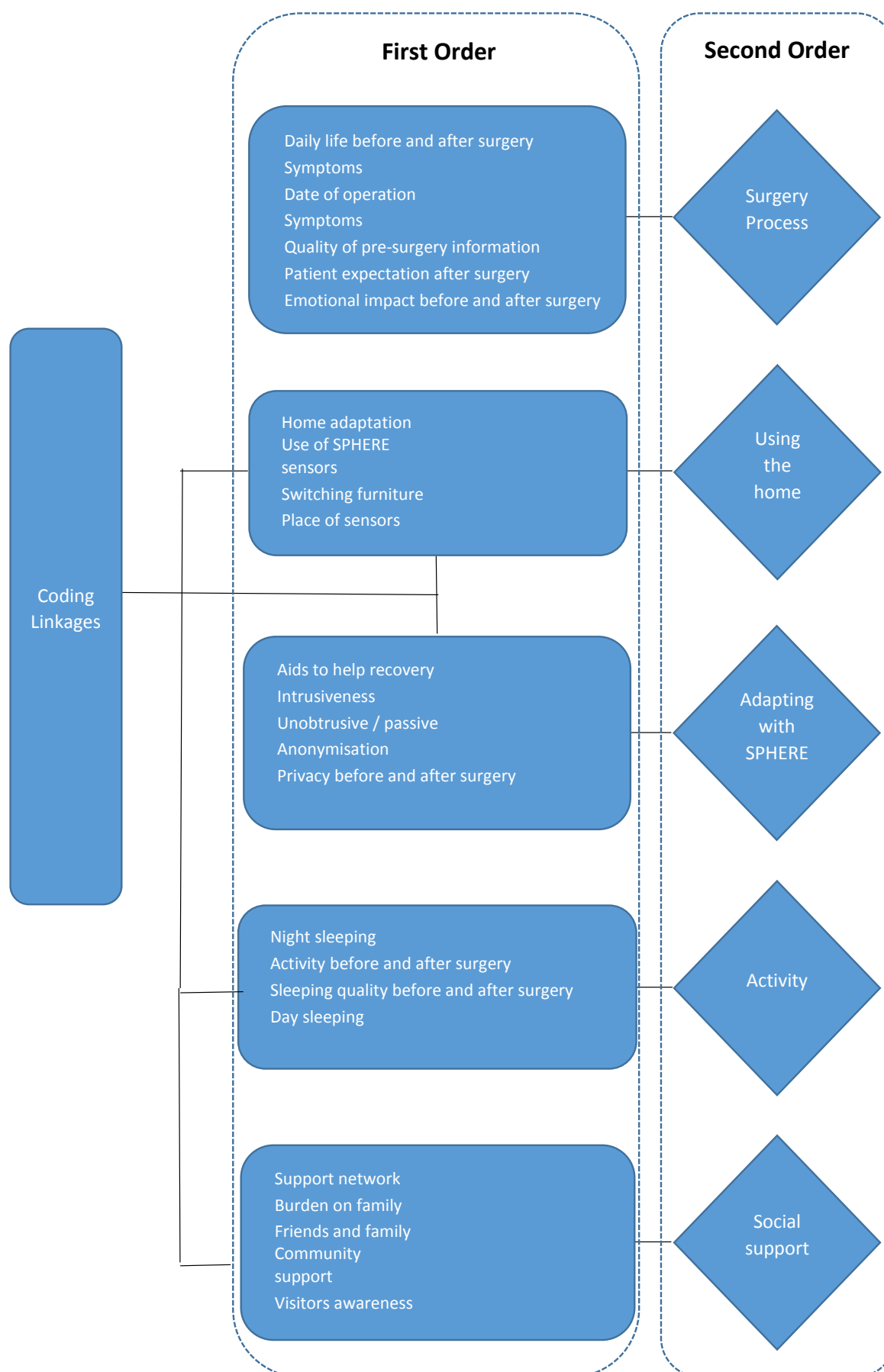

Supplement: Supplementary data [file bmjopen-2019-032205supp001.pdf]
